# The Mucosal Vaccine Adjuvant LT(R192G/L211A) or dmLT

**DOI:** 10.1128/mSphere.00215-18

**Published:** 2018-07-25

**Authors:** John D. Clements, Elizabeth B. Norton

**Affiliations:** a Department of Microbiology and Immunology, Tulane University School of Medicine, New Orleans, Louisiana, USA; UMKC School of Medicine

**Keywords:** adjuvant, dmLT, mucosal vaccines

## Abstract

Perhaps the best-studied mucosal adjuvants are the bacterially derived ADP-ribosylating enterotoxins. This adjuvant family includes heat-labile enterotoxin of Escherichia coli (LT), cholera toxin (CT), and mutants or subunits of LT and CT. These proteins promote a multifaceted antigen-specific response, including inflammatory Th1, Th2, Th17, cytotoxic T lymphocytes (CTLs), and antibodies. However, more uniquely among adjuvant classes, they induce antigen-specific IgA antibodies and long-lasting memory to coadministered antigens when delivered mucosally or even parenterally. The purpose of this minireview is to describe the general properties, history and creation, preclinical studies, clinical studies, mechanisms of action, and considerations for use of the most promising enterotoxin-based adjuvant to date, LT(R192G/L211A) or dmLT. This review is timely due to completed, ongoing, and planned clinical investigations of dmLT in multiple vaccine formulations by government, nonprofit, and industry groups in the United States and abroad.

## Abstract (precis)

Perhaps the best-studied mucosal adjuvants are the bacterially derived ADP-ribosylating enterotoxins. This adjuvant family includes heat-labile enterotoxin of Escherichia coli (LT), cholera toxin (CT), and mutants or subunits of LT and CT.

## INTRODUCTION

**FIG 1:**
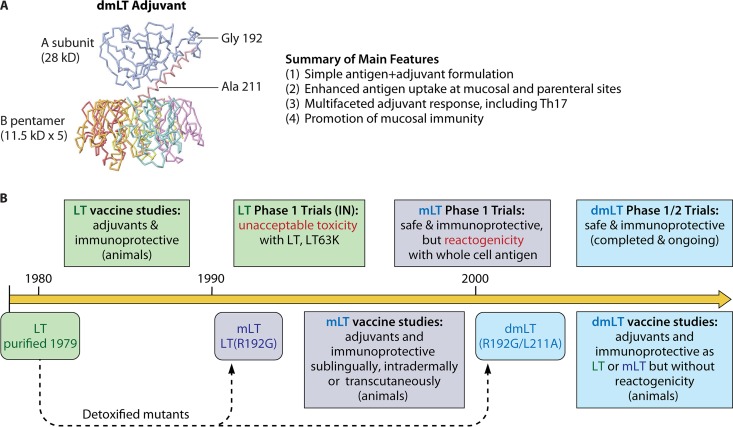
dmLT adjuvant structure, summary of main features, and creation timeline. See text for details.

The adjuvant dmLT, or more technically LT(R192G/L211A), is an 84-kDa polymeric protein with an AB_5_ structure composed of an enzymatically active A subunit (28 kDa) noncovalently associated with a pentameric B subunit (consisting of five 11.5-kDa monomers) as shown in Fig. 1A. dmLT is distinguished from its parent molecule heat-labile enterotoxin (LT) by the substitution of two residues in the A subunit, a glycine for an arginine at amino acid 192 (R192G) and an alanine for a leucine at amino acid 211 (L211A). The ribbon diagram of dmLT can be extrapolated from the crystal structure of the partially cleaved LT toxin (1, 2), although there may be as-yet-unresolved changes in three-dimensional (3D) structure due to the amino acid substitutions in the A subunit.

dmLT is an adjuvant that enhances vaccine-specific systemic and mucosal immune responses following mucosal or parenteral delivery, described in detail below (e.g., Table 1 and indicated references). These studies indicate four main features that define dmLT compared with other adjuvant systems.

(1) dmLT promotes immunity to antigens that are codelivered after simply admixing dmLT and the antigen in aqueous buffer. Thus, unlike many depot-type adjuvants, such as aluminum hydroxide, no advanced preparation or absorption is required to formulate the antigen/adjuvant vaccine. dmLT can be formulated with the antigen at either the point of manufacture or the point of delivery.
(2) Through the combined action of dmLT’s immunostimulatory properties and universal cell binding, uptake of codelivered antigens is enhanced and mucosal immunity is promoted. This enables the delivery of immunization formulations (most strikingly for subunit vaccines) at previously inaccessible sites, such as in oral (p.o.), sublingual (s.l.), transcutaneous (t.c.i.), etc., delivery. Many of these approaches are needle free and have the potential to increase ease of administration and compliance and lower the risk of disease outbreaks from unsafe injections (3–6).
(3) Unlike other adjuvants, such as aluminum hydroxide or many Toll-like receptor (TLR)-based adjuvants (e.g., monophosphoryl lipid A [MPL] and CpG), dmLT induces strong interleukin-17 (IL-17) recall cytokine secretion and antigen-specific Th17 responses after parenteral or mucosal immunization (7–15). This is a newly appreciated arm of the adaptive immune response that is critical in protection from pathogens, particularly in preventing infections in mucosal tissue and control of bacterial infections (16). In addition, IL-17 secretion enhances the availability of mucosal antibodies by upregulating polymeric Ig receptor levels in epithelial cells, increasing transport of secretory IgA (sIgA) into the lumen of mucosal tissue, and promoting T-independent B-cell differentiation into IgA-secreting cells (17–20).
(4) Last, dmLT promotes the development of mucosal immune responses following parenteral immunization (7, 21–23). While these observations are validated only in preclinical animal models thus far, this is a distinction from most vaccines delivered by parenteral injection, which can induce serum antibodies and cell-mediated immunity but only limited or nonexistent responses at mucosal surfaces. In the remaining sections, we will go into more detail on the history and creation of dmLT protein, preclinical studies, clinical studies, mechanisms of action, and considerations for use.

**TABLE 1:** Preclinical vaccine studies with dmLT^a^

Vaccine type and target pathogen	Antigen(s)	Route(s)	Model(s)	Major findings with adjuvant inclusion	Reference(s)
Subunit vaccines					
Clostridium difficile	PilA1, PilJ, PilW	p.o.	Mouse	NAO; low levels of antipilin antibodies and no protection from weight loss after oral challenge; no clear difference from other routes/adjuvant combinations	91
Clostridium difficile and *Shigella* species	Chimeric toxin A/toxin B; IpaB/IpaD	i.d., i.m., i.p.	Mouse	NAO; route and delivery comparison with microneedles: similar responses in dmLT-adjuvanted i.d. as alum-adjuvanted i.p. groups for antibody responses and response to C. difficile challenge; development of IgG and mucosal IgA after i.d. and i.m. vaccination with dmLT to *Shigella*	61
Clostridium tetani	Tetanus toxoid	p.o., i.n.	Mouse	Enhanced serum and mucosal antibodies, Th17 responses	1, 8
ETEC	EptA	i.d., p.o., s.l.	Mouse	NAO; route evaluation with adjuvanted vaccination: p.o., s.l., or i.d. delivery enhanced mucosal IgG and IgA; however, bacterial burden post-oral challenge reduced only with p.o. and s.l.; antibody avidity reduced in i.d. groups	92
Nontypeable *H. influenzae* otitis media	LB1, rsPilA, chimV4	t.c.i.	Chinchilla	Enhanced skin DC migration to lymphoid organs, serum IgG and IgA, and clearance of bacteria from nasopharynges/middle ears when combined with any antigen vaccine (prophylactic or therapeutic)	55
	chimV4	t.c.i.	Chinchilla	NAO; enhanced mucosal IgG, IgA, IFN-γ^+^, and IL-17 CD4 T cells; eradication of bacterial burden and biofilms in middle ears	22
	chimV4	t.c.i.	Chinchilla	NAO; enhanced antigen draining to lymphoid organs dependent upon therapeutic t.c.i. patch placement; enhanced Th1, Th17, ASCs, and mucosal IgM and IgG; reduced % of ears with otitis media and bacterial colonization observed with dmLT alone or in combination with antigen	11
Polymicrobial otitis media	rsPilA, chimV4, IHC	t.c.i.	Chinchilla	NAO; enhanced antigen-specific ASCs and serum IgG, IgM, and IgA antibodies; reduced incidence of polymicrobial (viral-bacterial) otitis media and disease time course	93
Helicobacter pylori	Lysate antigens, HpaA, UreB	p.o.	Mouse	Enhanced serum IgG, mucosal IgA, and splenic and stomach Th1 and Th17 responses; reduced bacterial colonization in stomach after oral challenge (equivalent to cholera toxin)	12
	Lysate antigens, freeze-dried	p.o., s.l.	Mouse	Increased activation of *ex vivo* DCs; enhanced Th1, Th17, and IL-17A^+^ IFN-γ^+^ CD4 T cells in DLN or stomach after s.l. but not p.o. (equivalent to cholera toxin)	13
Hepatitis B virus	HBsAg (bioencapsulated in maize)	p.o.	Mouse	Nonsignificant enhancement of serum IgG and IgA antibody responses	54
Vibrio cholerae	Polysaccharide conjugate (OSP-TT_hc_)	i.m.	Mouse	Enhanced level and vibriocidal activity of serum antibodies, formation of memory B cells, and survival by neonatal mice of lethal challenge	57
*Shigella* species	IpaB, IpaD, IpgC, IpaB/IpgC	i.n.	Mouse	NAO; antigen evaluation with adjuvanted i.n. vaccination: improvement of serum and mucosal IgG and IgA, ASCs, Th1 responses, and protection from i.n. challenge	94
	IpaB/IpaD	i.d.	Mouse	Enhanced level of serum antibodies, protection from lethal i.n. challenge (antigen dose dependent) with microneedle delivery	9
	IpaB/IpaD	i.d., s.l.	Mouse	NAO; route evaluation with adjuvanted vaccination: i.d. delivery enhanced mucosal IgG; s.l. delivery enhanced mucosal IgG and IgA; i.d. plus s.l. exhibited maximal mucosal IgG and IgA	23
	PSSP-1	i.n., i.d.	Mouse	NAO; enhanced protection to i.n. challenge with multiple *Shigella* species and serotypes after i.n. but not i.d. immunization	60
Whole-killed vaccines					
ETEC	Whole-killed plus LCTBA (ETVAX)	p.o.	Mouse	Enhanced serum IgG and fecal IgA antibodies (equivalent or better than CT)	59
Helicobacter pylori	Formalin inactivated, liquid or freeze-dried	p.o.	Mouse	No significant increase in serum IgG responses; however, after oral challenge, reduced stomach urease activity and stomach bacterial burden (equivalent to or better than mLT)	38
*Shigella* species	Formalin inactivated trivalent (S. flexneri 2a and 3a and Shigella sonnei)	i.n.	Mouse, guinea pig	In mice, no major adjuvant effect except with low antigen dose (IgA and protection from lethal i.n. challenge); in guinea pig, same or worse response to ocular challenge	58
Streptococcus pneumoniae	Chloroform-killed nonencapsulated strain RM200	b.c., s.l.	Mouse	Reduced bacterial burden after i.n. challenge and increased IL-17 secretion (comparison with nonadjuvanted groups performed with only mLT and CT adjuvants)	10
Poliovirus	Formalin inactivated trivalent (IPV)	s.l.	Mouse	Enhanced antigen dose-sparing, serum IgG, salivary IgA, and virus-neutralizing antibodies with thermoresponsive gel delivery system	56
		i.d., i.m.	Mouse	Enhanced germinal center markers, antigen dose sparing, serum IgG, fecal IgA, and virus-neutralizing antibodies	21
Live-attenuated vaccines					
Salmonella enteritidis	Strain JOL1087 ± secreted dmLT	i.m., p.o.	Chicken	Biological incorporation of dmLT into vaccine strain increased plasma IgG, intestinal sIgA, PBMC stimulation, and splenic cytokines (IFN-γ, IL-6, and IL-10) and reduced bacterial colonization after oral challenge	62, 90

^a^ Abbreviations: ASCs, antibody-secreting cells; DC, dendritic cell; DLN, draining lymph node; IHC, integration host factor; IPV, inactivated polio vaccine; NAO, no antigen-only group without dmLT.

## HISTORY OF LT-BASED PROTEINS AND dmLT CREATION

LT is expressed by enterotoxigenic Escherichia coli (ETEC) strains and was first purified by Clements and Finkelstein in 1979 (24). This effort differed from previous attempts to purify LT by conventional gel-filtration and ion-exchange chromatography as a result of their discovery that LT binds to galactose-containing gel filtration medium (e.g., agarose or immobilized d-galactose) and can be recovered by elution from columns following application of galactose-containing buffers. In addition, unlike cholera toxin (CT) from Vibrio cholerae (which had been purified in 1969), Clements and Finkelstein demonstrated that the majority of LT is not secreted from the bacterium *in vitro* but rather is in the periplasm in an unactivated form.

In the 1980s, CT was being used to investigate the intestinal IgA response, with Elson and Ealding elegantly demonstrating that CT can abrogate oral tolerance and promote serum IgG and mucosal IgA to p.o.-codelivered antigen (25, 26). Subsequent studies by Clements et al., published in 1988, demonstrated that LT could also prevent induction of oral tolerance and act as an oral adjuvant (27). In a prelude to the current understanding of tolerance and memory regulatory responses, they found that oral tolerance could be prevented if LT was included upon first exposure to an antigen but that tolerance could not be broken once established. In addition, enzymatic activity in the form of the A subunit (LTA) was required since recombinant LT B subunit (LTB) had no effect on induction of tolerance or oral adjuvanticity. A key component in the success and accuracy of this research was the use of recombinant proteins (e.g., LTB) as opposed to the products of dissociation chromatography using the holotoxin, the latter of which commonly resulted in CTB/LTB that was contaminated with a trace amount of holotoxin, which can complicate the interpretation of results in many older publications (28).

Despite contrary evidence in animal studies, clinical trials with human volunteers beginning in the 1990s demonstrated that even low doses (>2.5 µg) of p.o. LT induced diarrhea (29, 30). In parallel, Tamura et al. began testing native LT admixed with LTB as an intranasal (i.n.) adjuvant in humans (31). Subsequent animal studies by a variety of investigators showed excellent promotion of immune responses for multiple antigens delivered by this route. Initial success in clinical studies led to the licensure for an LT-adjuvanted inactivated influenza vaccine in Europe (Nasalflu; Berna Biotech), which was available in Switzerland for the 2000–2001 influenza season. Unfortunately, it soon became clear that the presence of LT was associated with cases of Bell’s palsy or facial paralysis in i.n. vaccine recipients, resulting in a 19-fold-higher risk of developing Bell’s palsy compared to case-controls in a retrospective study (32). Similar results were reported in phase 1 clinical trials with i.n. delivery of mutant LT(S63K) in adjuvanted HIV and tuberculosis subunit vaccines (33). Follow-up research suggests that the risk for Bell’s palsy after i.n. vaccination is a direct consequence of B-subunit neuronal ganglioside binding and retrograde axonal transport combined with the inflammatory response occurring with ADP-ribosyltransferase activity of intact AB_5_ proteins (34). While investigation into other administration routes continues, use of LT or any AB_5_ mutant protein for i.n. delivery in humans is unadvised based on these past safety issues (though our studies indicate that the enzymatically active A1 subunit [LTA1] free of any B subunit is a promising adjuvant for i.n. delivery [8]).

A number of investigators have genetically modified LT in an attempt to detoxify the molecule and make it safe for inclusion either as an antigen (in an ETEC vaccine) or for use as an adjuvant. Creation of dmLT was initiated through purposeful stepwise mutations of the LT holotoxin A subunit, based on our understanding of how the holotoxin interacts with mammalian cells. Induction of intestinal fluid secretion by LT and CT occurs after a series of events involving both changes to toxin structure and activation of intracellular signaling pathways, reviewed in reference 28. After B-subunit binding and entry, the subsequent proteolytic cleavage and disulfide bond reduction separate the A subunit into two domains: the enzymatically active A1 subunit and a smaller A2 peptide. Transport of A1 into the cytoplasm results in ADP-ribosylation of Gsα, followed by irreversible activation of adenylate cyclase and increases in intracellular levels of cAMP. In intestinal epithelial cells, this causes a dysregulation of cAMP-sensitive ion transport mechanisms which inhibits intracellular salt absorption, increases electrolyte transport into the gut lumen, and creates an osmotic gradient favoring intestinal water secretion (35).

Clements and Finkelstein had originally shown that proteolytic cleavage at position 192 was essential for activation of the LT molecule (24). Later in the early 1990s, Dickinson and Clements created LT(R192G), or mLT, by substituting a glycine for an arginine at position 192, thereby altering the proteolytically sensitive site in the A subunit that separates A1 and A2 and preventing trypsin cleavage and “activation” (36). In both *in vitro* assays and animal studies, mLT showed reduced toxicity (36, 37) but maintained adjuvanticity equivalent to LT, inducing a balanced Th1/Th2 cytokine and antibody subclass profile similar to native LT (10, 38–49). The success of these preclinical studies led to several clinical trials with mLT as an adjuvant. In a phase 1 escalating dose-safety study in adults, up to 50 µg of p.o. mLT given twice was well tolerated; however, 16.7% (2 of 12) of volunteers receiving a 100-µg mLT dose reported mild to moderate diarrhea (50). In contrast, >2.5 µg of native LT or CT causes diarrheal secretion in adult volunteers (29, 51). In other clinical studies, 25 µg of p.o. mLT alone was well tolerated, but when it was combined with killed whole-cell bacterial vaccines, 20% of adults receiving killed *Campylobacter* (52) or killed *Helicobacter* (53) p.o. vaccines experienced mild diarrhea. Thus, the combination of mLT and a whole-cell antigen delivered p.o. induced enough secretion to overcome the natural resorptive capacity of the intestine, resulting in the observed self-limited, mild diarrhea not seen with mLT alone.

In the early 2000s, in order to further detoxify mLT while preserving adjuvanticity, an additional mutation was added by changing leucine 211 to alanine (L211A) in the A1-A2 activation loop on the A2 domain. This resulted in the adjuvant dmLT. In a 2011 publication, we demonstrated that this extra mutation results in two significant differences: (i) dmLT is more sensitive to trypsin or intracellular proteolytic degradation (as opposed to activation) than either native LT or mLT, and (ii) 250 µg of dmLT completely lacks the ability to induce detectable intestinal secretion after p.o. feeding in a patent mouse assay compared with ≥5 µg LT or ≥125 µg mLT (1).

As summarized in Fig. 1B, dmLT is the product of more than 25 years of research on the use of bacterial ADP-ribosylating enterotoxins as adjuvants. While the adjuvant potential of LT and CT has been known for some time, reducing toxicity while preserving adjuvanticity was challenging. Our efforts to detoxify LT for use as an adjuvant through directed genetic mutations has yielded dmLT, whose mutations make it a subunit more vulnerable to intracellular proteolytic degradation but prevent cleavage into the highly enzymatically active A1 domain. However, dmLT maintains the immunostimulatory properties without associated epithelial cell cAMP intoxication or intestinal fluid secretion of the parent molecule LT or single mutant mLT.

## PRECLINICAL STUDIES

Since 2000, a number of preclinical vaccine studies have been published with dmLT adjuvant, as listed in Table 1. Of these, most target bacterial pathogens with subunit antigens, although some have examined dmLT-adjuvanted whole-killed or live-attenuated bacterial and viral vaccines. The target pathogens for many of these vaccine candidates cause predominantly gastrointestinal infections, including Clostridium difficile, ETEC, Helicobacter pylori, poliovirus, *Shigella*, *Salmonella*, and V. cholerae. However, other mucosal pathogens—nontypeable Haemophilus influenzae and Streptococcus pneumoniae—or organisms causing systemic infections—Clostridium tetani and hepatitis B virus—have also been evaluated. In addition, there are a large number of unpublished preclinical studies that have been performed by various groups leading to clinical trials.

We and other researchers have documented improvement of parenteral and/or mucosal immunity to bacterial and viral antigens following p.o., buccal (in the mouth by the cheek [b.c.]), s.l. (in the mouth under the tongue), i.n., t.c.i., intradermal (i.d.), and/or intramuscular (i.m.) delivery of dmLT-adjuvanted vaccination (8, 9, 22, 23, 38, 54–59). By far the most common findings after either parenteral or mucosal immunization were improvement of humoral and systemic immunity—including neutralizing antibodies and serum or mucosal IgA and Th17 responses—and protection from lethal challenge or bacterial colonization. Comparative evaluations verified that the adjuvant effects with dmLT are fairly equivalent to those with LT, mLT, or CT adjuvants, including Th17 responses to mucosal pneumococcal vaccine (10), serum IgG and fecal IgA to colonizing factors and LTB after p.o. immunization with an engineered whole-killed ETEC vaccine (59), tetanus toxoid-specific serum IgG and fecal IgA after p.o. or i.n. immunization (1, 8), anti-*Shigella* PSSP-1 serum IgG1 or IgG2a (60), and immunity and protection against H. pylori infection after s.l. immunization with lysate antigens (12) or p.o. immunization with formalin-inactivated bacteria (38).

Various delivery techniques or devices for i.d. delivery did not impair the adjuvant effect of dmLT, including the use of transdermal silk fibroin microneedles (61) and NanoPass MicronJet 600 needles (9). Similarly, various antigen forms—including freeze-dried forms (13, 38), polysaccharide conjugates (57), and even a live-attenuated strain modified to express dmLT (62)—also demonstrated improved immunity with inclusion of the adjuvant.

In all these studies, only three studies reported lack of significant immunity with dmLT adjuvant with specific formulations. The first was a study immunizing mice p.o. with germ-enriched maize material expressing hepatitis B antigen; however, slightly higher (but nonsignificant) antibody titers were observed in the dmLT-containing group, suggesting that more animals (to better power the study) or an optimized dose may be needed to see a clear adjuvant effect with this vaccine formulation (54). The second study evaluated doses of a highly immunogenic trivalent formalin-inactivated *Shigella* vaccine that saw improvement only in mucosal IgA and protection from lethal challenge in mice receiving dmLT admixed with the lowest-formulation dose (58). In this same vaccination study, dmLT provided no benefit in a guinea pig model of *Shigella* keratoconjunctivitis, which may indicate a failure of dmLT to boost eye-specific immunity. Last, in another study with *Shigella* IpaB/IpaD subunit vaccination, dmLT was unable to skew immunity to a nonprotective high dose of antigen, even though inclusion of dmLT at a medium dose elicited maximal protection from i.n. challenge (9). These studies reveal an important consideration in any vaccine formulation—there is a limit to the adjuvant effect. The antigen formulation, delivery dose, route, and likely other factors continue to play major roles in the overall outcome of responses postvaccination.

An interesting finding reported in several preclinical studies was that the dmLT-only control group provided some nonspecific protection from disease. A t.c.i. band-aid application of 10 µg dmLT significantly reduced the percentage of infected middle ears and bacterial burden of existing nontypeable *H. influenzae* biofilms in chinchillas despite any observable enhancement of antigen-specific cellular or humoral immunity (11). Similarly, fecal shedding after oral Campylobacter jejuni challenge was decreased (although the percentage of animals colonized was not) in mice who had been treated with three weekly doses of dmLT 1 week prior to infection (63). In this study, there was also no detection of antigen-specific immunity to C. jejuni. Last, mice receiving 5 µg dmLT i.n. on days 0 and 14 exhibited 64% survival of lethal Shigella flexneri 2a pulmonary challenge on day 21 (58).

In several studies, dmLT has been evaluated as a toxoid antigen for ETEC vaccines. dmLT can generate anti-LT antibody responses, detected routinely after p.o. or parenteral immunization (1, 9, 59, 63, 64). It has also been incorporated in a multiepitope fusion antigen for ETEC vaccination to neutralize activity of LT toxin (65). Of note, the existence of preexisting antibodies to dmLT does not impair the ability of subsequent immunization to provide adjuvant effects to a *de novo* antigen not previously seen by the immune system (E. B. Norton and J. D. Clements, unpublished data).

The consensus of these studies is that dmLT is uniquely able to induce systemic and mucosal responses after parenteral or mucosal immunization. The success of these studies has also led to recent preformulation studies evaluating buffers to promote stability and freeze-dried formulations to maximize dmLT adjuvant and/or antigen responses (66, 67). dmLT has also been used in immunologic studies for mucosal vaccination in order to evaluate how immunity is generated or impacted by infections (68, 69). In addition, as discussed in the next section, there are now several completed and planned clinical studies using dmLT.

## CLINICAL STUDIES

As shown in Table 2, dmLT has now been tested for safety and efficacy in several ongoing and recently completed human clinical trials. There have been two dmLT-only phase 1 safety studies. The first trial (NCT01147445) was a dose escalation study with a single dose of 5 to 100 µg p.o. dmLT. Treatment was well tolerated with no reported diarrhea, abdominal pain, anorexia, nausea, vomiting, or fever in any adult subject (70). The second dmLT-only phase 1 safety study (NCT02052934) was conducted to assess the safety and tolerability of dmLT when administered in three 1- to 50-µg s.l. doses compared with three 25-µg p.o. doses of dmLT in healthy adult subjects. This study also assessed long-term safety through 7 months postvaccination. While this study has concluded, no results have been reported to date. An additional phase 1 safety trial (NCT02531685) with dmLT i.d. administration is currently recruiting. The primary objective of this study is to assess the safety and tolerability of dmLT when administered in three i.d. injections over a range of dosages in healthy adult subjects.

**TABLE 2:** Completed and ongoing clinical trials with dmLT adjuvant^a^

Pathogen: antigen	Route	Study design (Clinicaltrials.gov ID [reference {if available}])	Population; status: results
None: none	p.o.	Phase 1, escalating dose safety study (NCT01147445 [70])	U.S. adults; completed: no detectable SAE
ETEC: whole-killed (ETVAX)	p.o.	Phase 1, whole cells ± 10 or 25 µg dmLT (EudraCT no. 2011-003228-11 [71])	Swedish adults; completed: 10 µg dmLT enhanced responses to less immunogenic antigens
ETEC: live-attenuated (ACE527)	p.o.	Phase 1 and 2b, 25 µg dmLT with live-attenuated ETEC (NCT01739231 [72])	U.S. adults; completed: dmLT enhanced protective efficacy from oral challenge 6–7 mo postimmunization
None: none	s.l.	Phase 1, escalating dose safety study (NCT02052934)	U.S. adults; completed
None: none	i.d.	Phase 1, escalating dose of 0.1, 0.3, 1, or 2 µg dmLT (NCT02531685)	U.S. adults; recruiting
ETEC: whole-killed (ETVAX)	p.o.	Phase 1 and 2 escalating dose of ETVAX ± 2.5, 5, or 10 µg dmLT (NCT02531802)	Bangladesh infants, toddlers, children, adults; completed
ETEC: subunit (CS6)	i.m.	Phase 1, escalating dose safety study (NCT03404674)	U.S. adults; recruiting
ETEC: whole-killed (ETVAX)	p.o.	Phase 2b, ETVAX plus 10 µg dmLT (EudraCT no. 2016-002690-35)	Finnish adult travelers to Benin; ongoing

^a^ Abbreviations: SAE, severe adverse events; ID, identifier.

There have also been a number of phase 1 and 2 trials with dmLT as one component of killed or live-attenuated whole-cell ETEC vaccines that are either completed or ongoing. The killed whole-cell vaccine (ETVAX) is a formalin-inactivated, recombinant E. coli expressing increased levels of ETEC colonization factors (CFs) combined with a recombinant protein (LCTBA), which is a hybrid between the binding subunits of LTB and CT B subunit. The first of these studies (EudraCT no. 2011-003228-11) evaluated the safety of ETVAX administered alone or combined with 10 µg or 25 µg dmLT in two p.o. doses (71). This study observed only minor adverse events and reported no significant differences in adverse events between vaccination groups with and those without dmLT. The vaccine response generated was potent even in the nonadjuvanted group; however, 10 µg dmLT boosted immunity to the least immunogenic antigen evaluated, CS6, and increased the percentage of IgA responders to all five antigens in the vaccine strain to 83% from 74%. In this study, the 10-µg dose of dmLT was optimal. Since none of these studies were performed without the 1-mg dose of LCTBA, the influence of a 25- to 100-fold excess of a B-subunit antigen that competes for receptor binding with dmLT cannot be determined.

A follow-up phase 1 and 2 double-blind, placebo-controlled, dose-escalation study (NCT02531802) evaluating the safety, tolerability, and immunogenicity of ETVAX alone and together with dmLT in descending age groups (45 years to 6 months) was conducted in Bangladesh. This study observed some vomiting in young children when a full adult dose was administered, but the vaccine was tolerable at fractional doses with no significant differences in adverse events in vaccine groups with or without dmLT (F. Qadri, M. Chowdhury, T. Bhuiyan, M. Akhtar, F. Khanam, T. Ahmed, A. Lundgren, L. Bourgeois, R. Walker, N. Maier, A. Fix, T. Wierzba, and A. Svennerholm, presented at the Second International Conference on Vaccines for *Shigella* and ETEC [VASE], Mexico City, Mexico, 13 to 15 June 2018). An immunologic assessment is under way. The same vaccine (ETVAX plus 10 µg dmLT) is currently being evaluated in a phase 2b trial (EudraCT no. 2016-002690-35) to evaluate safety, immunogenicity, and diagnostic methodology and estimate vaccine efficacy of an oral ETEC vaccine for prevention of clinically significant ETEC diarrhea in healthy adult travelers visiting West Africa.

The use of dmLT with a live-attenuated ETEC vaccine, ACE527, was evaluated in a phase 1 and 2b challenge trial (NCT01739231). For this study, individuals were immunized three times p.o. with ACE527, an admixture of three frozen or lyophilized attenuated ETEC strains expressing CFA/I, CS1, CS2, CS3, CS5, CS6, and LTB, with or without 25 µg dmLT. Six to seven months after the last immunization, individuals were orally challenged with ETEC strain H10407 and monitored for moderate to severe diarrhea (MSD), stool volume, number of stools, and shedding of the challenge strain. Significantly, the protective efficacy of the vaccine against MSD in individuals immunized with ACE527 plus dmLT was 65.9%, compared to 20% in individuals receiving ACE527 alone (72). Addition of dmLT also increased the protective efficacy against diarrhea of any severity to 58.5% compared to −3.7% in individuals receiving ACE527 alone, reduced the mean stool volume (30 g versus 859 g) and total number of stools (1 versus 13), and produced a 13-fold reduction in shedding of the challenge strain compared to individuals receiving ACE527 alone. The mechanism of protection engendered by inclusion of dmLT in the formulation is not clearly defined, but studies to evaluate this are under way.

Recruitment is under way for one additional ETEC vaccine trial (NCT03404674). This will be a phase 1 open-label, dose-escalating study of 5 to 45 µg of a prototype CS6 vaccine (CssBA) administered three times i.m. with and without 0.1 to 0.5 µg of dmLT. The purpose will be to examine reactogenicity of the antigen, adjuvant, and formulation when administered i.m. This will be the first use of dmLT i.m. in humans.

These clinical studies indicate that dmLT is safe and efficacious. More information will become available as study results are reported in the future.

## MECHANISM OF ACTION

The broad mechanisms of action for LT, CT, and related proteins have been well established over the past decades and extensively reviewed (28, 73), and slight but significant differences in the immunologic biases between CT- and LT-based adjuvants have consistently been reported in the literature (e.g., references 28 and 74). Still, the subunits of these AB_5_ proteins contribute uniquely to their mechanisms of action. The B subunit is responsible for receptor binding, leading to cellular entry, and, during mucosal delivery, helping shuttle whole-vaccine antigens across mucosal surfaces (75). The A subunit binds to cytosolic proteins (e.g., ADP-ribosylation factors [ARFs]) and ADP-ribosylates Gsα, resulting in irreversible adenylate cyclase activation and accumulation of intracellular cAMP. The complete immunologic effects of LT appear to require the B subunit, A subunit, and some level of cAMP induction. LT promotes *in vitro* dendritic cell activation, cytokine secretion, and Th17 cell induction through processes that can be mimicked with cAMP analogs or other cAMP-inducing agents like forskolin (75, 76). However, when isolated B subunit (e.g., LTB) or enzymatically inactive mutants of LT are substituted for native LT as adjuvants, reduced or ablated immunologic effects are observed (37, 77–79). In recent studies, we have demonstrated that purified A subunit of LT has adjuvant properties by itself, inducing a similar quality (e.g., mixed Th1/Th2/Th17) but smaller magnitude of immune responses than native LT, whereas the B subunit alone induces a more Th2/T regulatory cell (Treg)-skewed response (8).

A major question with these adjuvants has always been whether toxicity can be divorced from adjuvanticity. With dmLT, we have determined that detoxification occurs without a reduction in adjuvanticity due to cell-specific effects. dmLT treatment results in no detectable intestinal secretion and over 1,000 times less cAMP in cultured epithelial cells compared to native LT (1), similar to the difference in secretion observed in human volunteers after p.o. treatment with LT versus dmLT (29, 30, 70). In contrast, during comparative vaccine studies dmLT exhibits adjuvanted immunity equivalent to that of LT, mLT, or CT (1, 8, 10, 12, 38, 60). In our newer experiments with murine dendritic cells (DCs), key initiating cells of the immune system, there are surprisingly few differences between cAMP levels, activation, cytokine secretion, and expansion of CD4 T cells between LT, mLT, and dmLT adjuvants (unpublished data). Thus, detoxification of LT into dmLT results in a cellular specific change in the mechanisms of action on epithelial cells responsible for secretion but not DCs responsible for immune stimulation.

**FIG 2:**
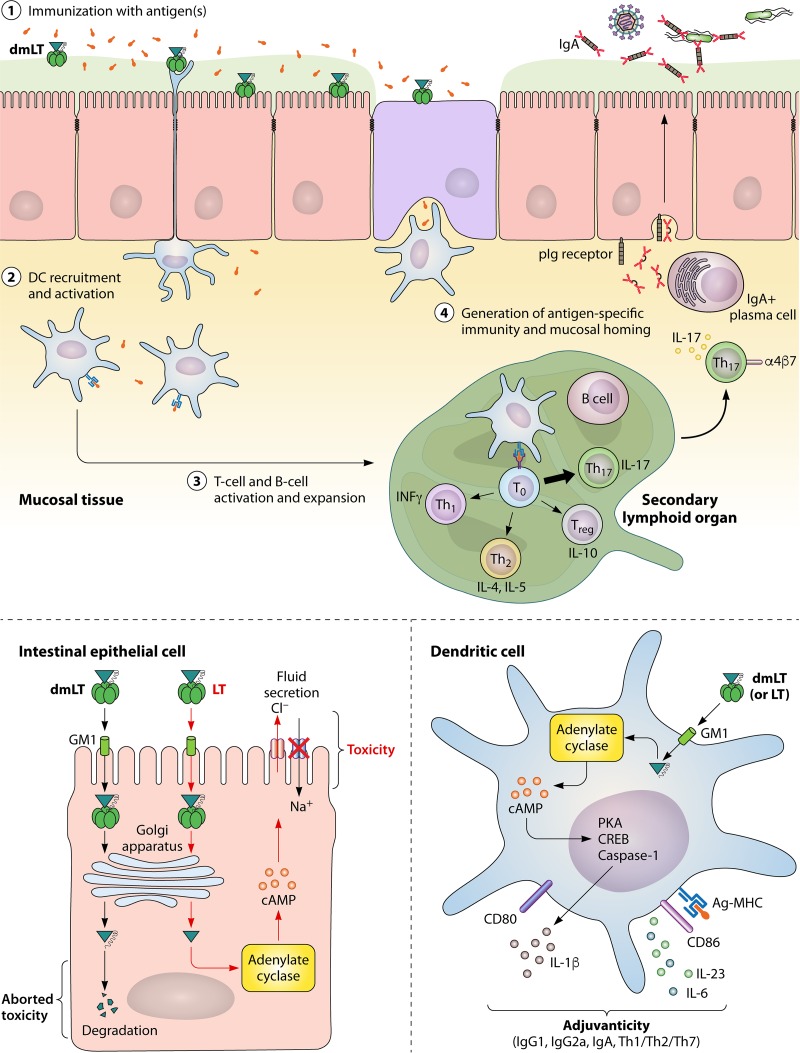
Mechanisms of the adjuvant dmLT. See text for details.

The immunologic events that occur with dmLT adjuvant can be summed up in the steps depicted in Fig. 2 (with supporting evidence also described below). First, at the site of immunization, antigen uptake and activation of innate immunity are promoted, including cytokine/chemokine (IL-8, granulocyte colony-stimulating factor [G-CSF]) production by epithelial cells. Second, DCs are recruited to the site of immunization and activated for antigen processing/presentation (including antigen loading of major histocompatibility complex [Ag-MHC]), upregulation of costimulatory molecules (CD80 and CD86), and polarizing cytokine secretion (IL-1, IL-23, IL-6, and G-CSF). Third, these adjuvant-activated and antigen-loaded DCs drain to secondary lymphoid organs and mediate production of antigen-specific T-helper (Th) cells and B-cell differentiation into IgA/IgG antibody-secreting plasma cells (PC). A mixed Th1/Th2/Th17 response is induced after immunization, with particularly strong induction of Th17 cells (1, 10, 15, 69) and mucosal homing markers (7). Th17 cells are recognized as integral in immunity, including promoting germinal center formation in secondary lymphoid organs and enhancing IgA secretion (18–20).

The role for DCs as a central orchestrator for immunity has strong scientific evidence. *Ex vivo* treatment of small intestinal explants with dibutyryl cAMP, mLT, LT, or CT results in recruitment of DCs to lymphoid follicle-associated epithelia for enhanced luminal antigen uptake (75). Depletion of DCs prior to p.o. CT administration inhibits generation of adjuvant-associated CD4 T-cell and antibody responses unless much higher doses of antigen are used (80). Other studies have shown that DC treatment with LT and other cAMP stimulants (including dibutyryl cAMP, forskolin, and phosphodiesterase inhibitors) increases chemokine expression (81), upregulation of maturation markers (76, 82, 83), and antigen presentation for T-cell responses, including Th2 (84, 85), Th1 (86), and Th17 (8, 10, 83, 87, 88). Similarly, after i.d. microneedle injection with dmLT-adjuvanted vaccine, murine CD11c^+^ DCs in the skin were shown to take up *Shigella* subunit antigens as soon as 4 h postimmunization (although other antigen-presenting cells, including neutrophils, macrophages, and Langerhans cells, also took up antigen) (9). In a chinchilla model of nontypeable *H. influenzae* otitis media, carboxyfluorescein succinimidyl ester (CFSE)-labeled dermal DCs (DC-SIGN^+^ CD11c^+^ CD207^−^) were observed in the nasal-associated lymphoid tissue (NALT) after t.c.i. immunization with dmLT and antigen (55). In another paper by this same group, the authors were able to detect labeled dmLT and antigen in CD11c^+^ secondary lymphoid organs that depended upon the placement of a t.c.i. band-aid vaccine (22).

The activation of DCs with LT and dmLT triggers a strong Th17-biased response due to activation of caspase 1 inflammasome and subsequent secretion of specific secreted factors, including IL-1 and IL-23. Brereton et al. elegantly showed that mouse dendritic cells stimulated with LT secrete IL-1β that is required for generation of Th17 cells and can be inhibited with pretreatment with caspase 1 and NLRP3 inflammasome chemical inhibitors (83). Similarly, restimulation of human peripheral blood mononuclear cells (PBMCs) from recently vaccinated individuals with dmLT and vaccine antigens enhanced IL-17A and IL-13 secretion that was prevented when cultures included anti-IL-1β and anti-IL-23 neutralizing antibodies (15). In a follow-up study, these authors demonstrated that dmLT-stimulated PBMC cytokine expression could be detected through intracellular staining of CD4 T cells and that this adjuvant effect on PBMCs or isolated monocytes could be prevented with inhibition of protein kinase A (PKA), IL-1RA (soluble IL-1 receptor), or caspase 1 (14). The consequences of this antigen-presenting cell (APC) stimulation are immune activation and promotion of mucosal and systemic immunity. Th17 induction and IgA isotype class switching are also important mediators of the effect of dmLT. In addition, using tetramer CD4 T-cell studies in mice, Frederick et al. have also shown upregulation of mucosal homing markers α4β7 on lymphocytes in draining lymph nodes after i.d. or i.m. immunization and corresponding localization of T cells within intestinal tissue (7). This indicates that something in the adjuvant-DC interaction promotes upregulation of gut homing markers, although at this point the exact mechanism is unclear.

Intriguingly, as reported above, several studies now indicate that dmLT can also provide nonspecific protection from disease (11, 58, 63). These reports are similar to older studies done by us and others with related proteins. For example, we have shown that 5 µg CT or mLT improves survival and decreases weight loss from influenza virus pulmonary challenge 24 h after a single or double weekly i.n. treatment (74). Improved survival with this regimen correlated with appearance of inducible bronchus-associated tissue (iBALT) structures in the lung. Similarly, a lower trend of lung CFU after Streptococcus pneumoniae nasopharyngeal challenge was observed in mice who had received their last boost of CT or mLT by i.n. immunization 1 month earlier (10). These studies indicate that activation of innate immunity is likely occurring to mediate these effects. Whether these represent long-term or epigenetic changes like those observed with trained immunity (89) is still unclear and requires further study.

Last, it is important to note that responses generated with dmLT may not be equivalent to all enterotoxins, particularly when specific mechanisms or secretion of specific cytokines is being measured (12, 14). For example, dmLT alteration of gene expression in murine splenic CD11c^+^ DCs only partially overlaps those of CT, including some notable differences in TSP-1, IL-6, MIP-2α (higher with CT), and gamma interferon (IFN-γ) (higher with dmLT) (12). These slight variations may have important considerations in generation of protective immunity.

In conclusion, studies to date indicate that a precise series of events takes place with dmLT stimulation that results in a protein that cannot induce enterocyte intoxication but is a potent stimulator of APCs and vaccine immunity. Future research into the precise intracellular mechanisms responsible for these adjuvant-induced responses is warranted.

## CONSIDERATIONS FOR USE

dmLT adjuvant has the unique potential to promote protective, long-lasting immunity to vaccine antigens when included in vaccination. However, as with any aspect of a vaccine formulation there are several caveats of use that should be carefully scrutinized.

(1) **Is dmLT the best adjuvant for anticipated delivery route?** dmLT is a good adjuvant to promote dose sparing, mucosal immunity, and/or mucosal delivery. In the last characteristic, dmLT is one of the few adjuvants that can directly assist antigen uptake at mucosal sites through the activity of its B subunit. Current data indicate that dmLT is safe and efficacious in promoting antigen responses with s.l. and p.o. routes but should not be used i.n. Less information is available for i.d. and i.m. routes, but ongoing clinical trials will determine this and also provide clinical data that dmLT may be able to safely promote mucosal immunity even with parenteral delivery routes.
(2) **Is dmLT the best adjuvant to promote immunologic biases associated with antigen-specific disease protection?** dmLT adjuvant enhances a mixed Th1/Th2/Th17 cellular response and enhances magnitude and longevity of antibody responses. However, the specific outcome of any adjuvanted vaccine responses will be heavily influenced by the nature of the antigen itself. This has been demonstrated recently by Leach et al., examining IL-17 secretion in human cells restimulated with human vaccine antigens and dmLT (15). Immunologic outcomes will need to be evaluated for each antigen(s), which may be complicated by delivery route, dose of antigen or adjuvant, and interactions between vaccine components (e.g., TLR ligands).
(3) **Which dose of dmLT adjuvant should be used?** Route studies have demonstrated clear differences in doses of dmLT required by each route. For example, dmLT or similar adjuvants are commonly used at a 10- to 25-µg range p.o., 1 to 5 µg s.l., 10 to 50 µg t.c.i., or 0.1 to 5 µg parenterally. However, many studies have indicated that these adjuvant doses may depend upon the nature of the antigen (e.g., subunit, whole-killed, or live-attenuated), as the combination with inflammation induced by antigen alone may play a significant role in immunologic outcomes. Basic immunology studies, furthermore, have clearly indicated that “more is not always better.” Too much inflammation or high doses of antigen can suppress the magnitude of responses and also bias to a stronger antibody/Th2 response. This appeared to be the case in ETVAX clinical trials, where 10 µg p.o. dmLT was superior to 25 µg (71). We also observed higher induction of antibodies and lower development of Th1/Th17 memory responses in mice immunized i.m. or i.d. with 1 µg dmLT and a Mycobacterium tuberculosis subunit antigen at 5 µg compared with 0.5 µg antigen (Norton and Clements, unpublished).
(4) **Potential differences between human and animal models.** The enterotoxin family of adjuvants has been successfully used in fish, avian, rodent, rabbit, pig, monkey, and human species (e.g., reference 90), often at relatively similar doses between species, pointing to their versatility as an adjuvant family. However, there may be species differences in receptor-ligand interactions or other host cell factors related to dmLT and possibly those pertinent to other vaccine antigens. Therefore, one should be careful in extrapolating or assuming that vaccine formulation research in animal models will perfectly mimic human responses.

## CONCLUSION AND FUTURE DIRECTIONS

In conclusion, dmLT is the product of more than 25 years of research. It was the purpose of this review to succinctly describe the history of dmLT and all published studies with dmLT, thereby providing the first review on this adjuvant protein. Both preclinical and clinical data thus far indicate that dmLT is a safe and effective adjuvant that in the right formulation can promote protective immunity. Unanswered questions for future studies include limitation on use of dmLT adjuvant, safety of antigen-adjuvant combinations, and formulation optimizations (including for route and non-cold-chain delivery, such as stability buffers). In addition, the mechanisms of adjuvanticity and generation of protective immunologic outcomes with dmLT adjuvant require more detailed study. Similarly, comparisons of dmLT with older enterotoxin adjuvants (e.g., LTK63 or LTR72) or other adjuvant classes (e.g., alum and TLR agonists) or even in combinatorial adjuvant formulations are warranted.
